# Supplementary material for: Spectro-temporal encoded multiphoton microscopy and fluorescence lifetime imaging at kilohertz frame-rates
Source: Nat Commun. 2020 Apr 28;11:2062. doi: 10.1038/s41467-020-15618-w (PMC7188897; doi:10.1038/s41467-020-15618-w)
Supplement: Supplementary file 1 — Supplementary Information [file 41467_2020_15618_MOESM1_ESM.pdf]

Supplementary Information

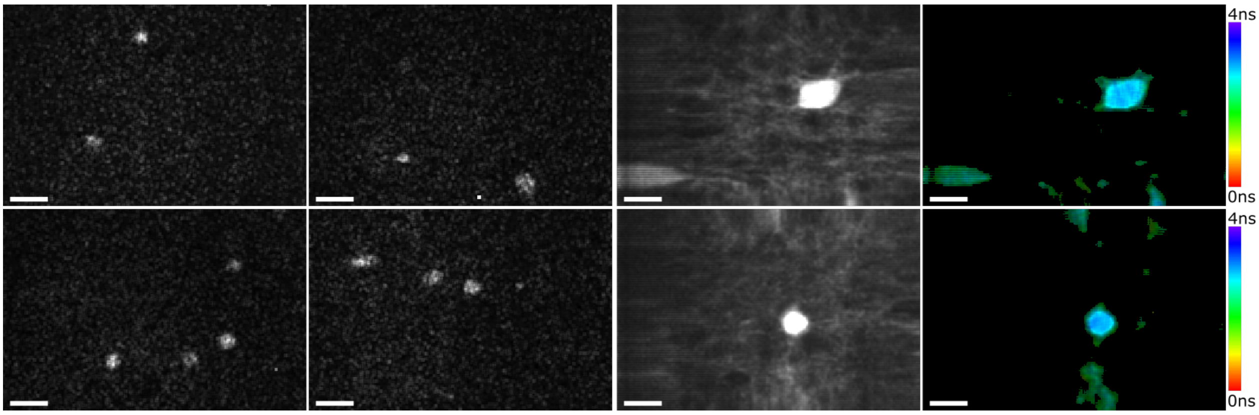

Supplementary Figure 1 Kilohertz frame-rate Imaging of fluorescent proteins-in *ex vivo* mouse brain in layer 2/3 at a depth of ~150-250 µm below the dura. The mouse was genetically modified to express the fluorescent protein tdTomato (see methods). The four images on the left were taken at a frame-rate of 2kHz and 2-times averaged (i.e. 1ms recording time). The images were spatially binned with 3x3 pixels as only single photons were detected per focal volume (cf. supplementary figure 2). Individual neurons are visible, however low SNR is achieved at these high frame-rates as fluorophore levels were too low and the fluorescent signal was already saturated. The pictures on the right were averaged 200-times, which leads to sufficiently high photon numbers for FLIM (right, last column). Each image pair on the right was acquired within 100ms recording time. Power after the objective was 150mW, i.e. 26W pulse peak power.

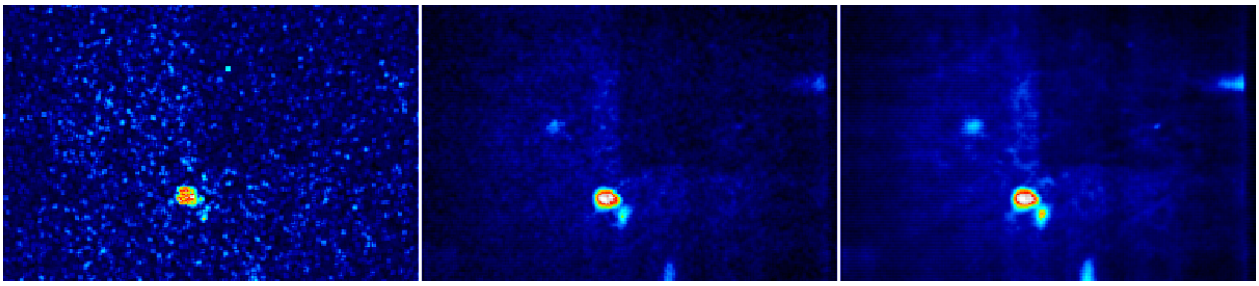

Supplementary Figure 2 Comparison of images between averaging 2-times (left), 200-times (middle) and 2000-times (right). The acquisition times are thus 1ms, 100ms, and 1000ms, respectively. More averaging leads to much increased SNR (see supplementary figure 3) and also permits extracting the lifetime of the tdTomato fluorescence (see supplementary figure 4). On the upper right corner there is a photobleached square visible. This bleaching was caused by the previous imaging position where the sample was imaged for ~10 minutes. Power after the objective was 150mW, i.e. 26W pulse peak power.

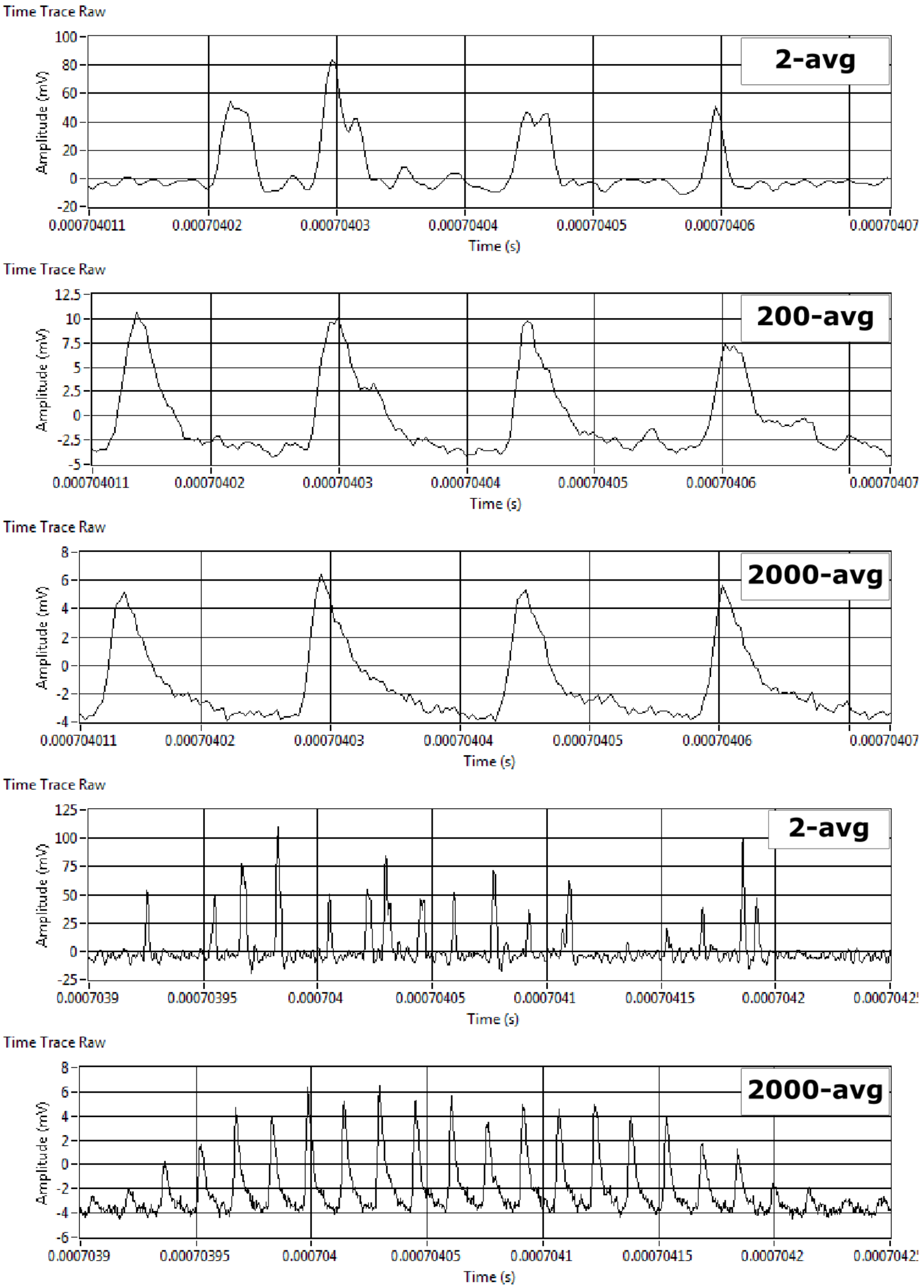

Supplementary Figure 3 Time Traces for the raw fluorescence signal from a tdTomato fluorescing neuron from supplementary figure 2. The 2-times averaged time trace shows individual photons arriving at the detector. In the first image, 8 photons are visible, where each photon generates an amplitude of 50mV and has a temporal width of 1026ps corresponding to the IRF (cf. Fig. 2). The sparsity of the excited fluorescent photons explains the low SNR of the 2-times averaged images in supplementary figures 1 and 2. Only when averaging many frames (200 and 2000-times averaging shown), the ensemble behavior of the fluorescent lifetime decay becomes visible. Thus, for this low concentration of

tdTomato fluorescent proteins in the genetically-modified mouse brain the imaging speed of SLIDE of 2 kHz cannot be reached at sufficiently high SNR to permit ΔF/F0 determination of action potentials.

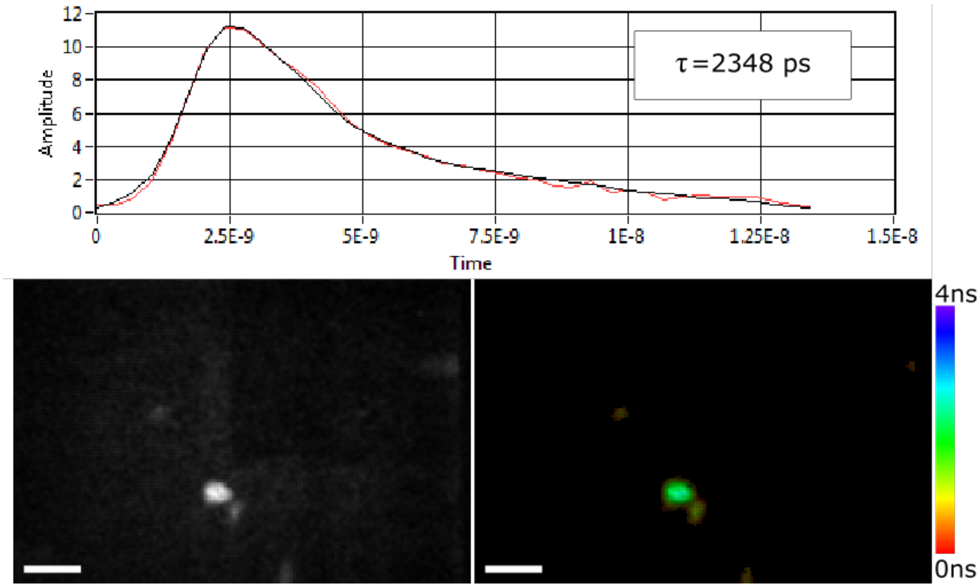

Supplementary Figure 4 SLIDE images of tdTomato expressing neurons. The lower images represent the TPM image (left) and the FLIM image (right). The frame-rate was 2kHz and 200 images were averaged to reach sufficient photon levels for reliable mono-exponential lifetime fits. The decay data together with a monoexponential, convoluted lifetime fit for a single pixel is shown above yielding a lifetime of 2348ps. The TPM image was 3x3 spatially binned, the FLIM image was 7x7 spatially binned. Scale bars represents 10µm. The total acquisition time for the image pair was 100ms, so neuronal imaging with FLIM capability at 10Hz imaging rate seems reasonable with the SLIDE system.

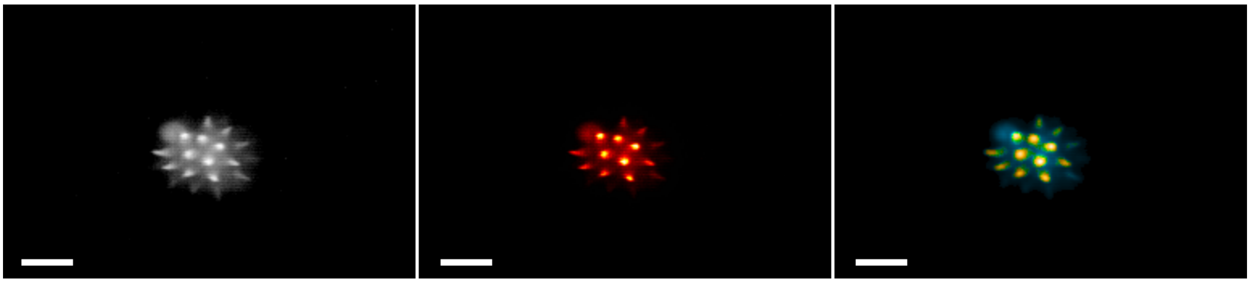

Supplementary Figure 5 Two-Photon Fluorescence and TP-FLIM Images of a Pollen grain. The image to the left is the fluorescence image displayed on a logarithmic scale. The spikey protrusions are on the sub µm scale and nicely visualize the high spatial resolution of the SLIDE microscope. The middle image is the same image with a "Red Hot" lookup table on a linear scale. The image on the right is the FLIM image where the spikes show a different fluorescent lifetime than the pollen body. The images were 100-times averaged, corresponding to 20Hz video-rate acquisition time for these images. Scale bars represent 10µm.

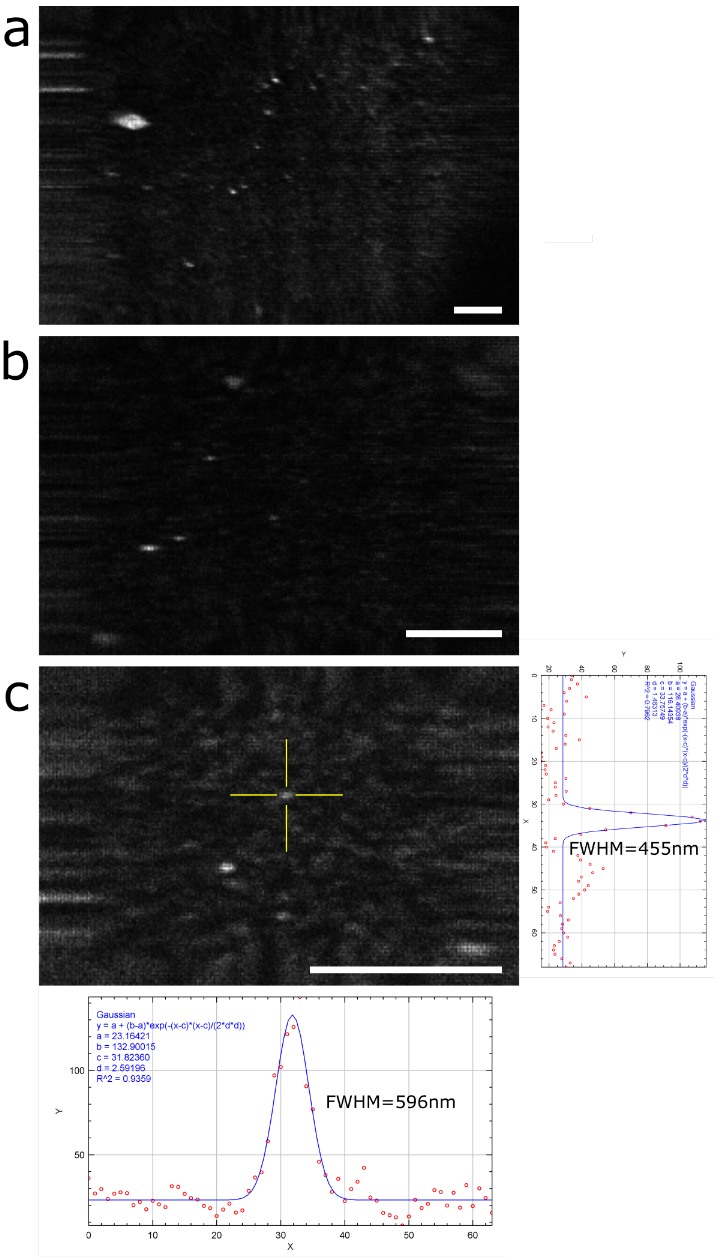

Supplementary Figure 6 Lateral Resolution measurement using 100nm fluorescent beads. Figure a shows the image with full span of the FDML laser, thus covering 100µm FOV in x-direction (spectral scanning direction). Scale bars represent 10µm, images were 100-times averaged. Figure b shows a zoomed-in version where the FDML span was adjusted to half its span by lowering the driving voltage by a factor of two. Figure c again uses half the FDML voltage, thus only covering 25µm FOV sampled by 256 pixels. The y-axis was also zoomed in and covers 22.5µm using 170 pixels in subfigure c. The line plots along the yellow lines in Fig. c together with gaussian fits are shown next to the images. The fits yield a FWHM resolution of 596nm and 455nm in x- and y-direction, respectively. The calculated resolution is 556nm for the x-axis (spectral resolution, see methods) and 380nm for the y-axis (diffraction-limit), so resolution close to the expected values is achieved.
